# Stable Rates of Obstructive Hypertrophic Cardiomyopathy in a Contemporary Era

**DOI:** 10.3389/fcvm.2021.765876

**Published:** 2022-01-06

**Authors:** Michael Butzner, Douglas L. Leslie, Yendelela Cuffee, Christopher S. Hollenbeak, Christopher Sciamanna, Theodore Abraham

**Affiliations:** ^1^Department of Public Health Sciences, Pennsylvania State College of Medicine, Hershey, PA, United States; ^2^Program in Epidemiology, University of Delaware College of Health Sciences, Newark, DE, United States; ^3^Department of Health Policy and Administration, Pennsylvania State University, University Park, PA, United States; ^4^Department of Medicine, Pennsylvania State College of Medicine, Hershey, PA, United States; ^5^Hypertrophic Cardiomyopathy Center of Excellence, University of California, San Francisco, San Francisco, CA, United States

**Keywords:** obstructive hypertrophic cardiomyopathy, hypertrophic cardiomyopathy, prevalence, claims data, sex, age

## Abstract

Hypertrophic cardiomyopathy is the most common genetic heart disease in the US, with an estimated prevalence of 1 in 500. However, the extent to which obstructive hypertrophic cardiomyopathy is clinically recognized is not well-established. Therefore, the objective of this study was to estimate the annual prevalence of clinically diagnosed oHCM in the US from 2016 to 2018. Data from the MarketScan® database were queried from years 2016 to 2018 to identify patients with ≥1 claim of oHCM (International Statistical Classification of Disease and Related Health Problems diagnosis code: I42.1). Prevalence rates for oHCM were calculated and stratified by sex and age. In 2016, 4,612 unique patients had clinical diagnosis of oHCM, resulting in an estimated oHCM prevalence of 1.65 per 10,000. The prevalence of oHCM in males and females was 2.07 and 1.26, respectively. Prevalence of oHCM was highest in patients 55–64 years of age (4.82). Prevalence of oHCM generally increased with age, from 0.36 per 10,000 in those under 18 to 4.82 per 10,000 in those 55–65. Trends in prevalence of oHCM over time, including by sex and age group, remained similar and consistent in 2017 and 2018. The prevalence of oHCM was stable over the 3-year time period, including higher rates of oHCM in males and patients aged 55–64 years. These results suggest that the majority of privately insured patients with oHCM are undiagnosed in the US and reinforce the need for policies and research to improve the clinical identification of oHCM patients in the US.

## Introduction

Hypertrophic cardiomyopathy (HCM) is the most common genetic heart disease in the United States (US), with an estimated prevalence of 1 in 500 persons from echocardiography-based studies (1). Patients with HCM are at risk for heart failure, stroke and cardiac arrhythmias, including sudden cardiac death (2). Among patients with HCM, approximately two thirds have obstruction (oHCM) either with rest or with provocation (3). Obstruction has been considered the primary cause of symptoms for patients and is associated with worse clinical outcomes (3, 4). In 2013, the HCM diagnosis rate was estimated at 1 in 3,000 persons (0.03%) in the US, based on an analysis of private practitioner and healthcare system medical claims (5). Maron et al. (5) identified HCM in about 100,000 patients, but it is estimated that 700,000 people in the US may be affected by HCM (1, 5). These existing studies show that the majority of HCM patients in the US are clinically unrecognized and the number of diagnosed HCM patients is significantly lower than the estimated prevalence of HCM in the general population (5).

However, these previous studies in the US have reported on the prevalence of HCM in its entirety and have not specified prevalence rates of patients with obstruction. Patients with obstruction may benefit from guideline directed medical therapy including beta-blockers, calcium channel blockers, and disopyramide (6, 7); and in the future, from emerging medical therapies currently being tested in clinical trials (8–11). Focusing on oHCM prevalence in the US will allow us to estimate the number of patients that are symptomatic and require these contemporary treatments by experienced referral centers. A recent study analyzing obstruction in patients that underwent cardiac magnetic resonance imaging from the United Kingdom (UK) Biobank (UKBB) reported a prevalence of oHCM of 1 in 517 persons (0.19%) (12). Similarly, we sought to close the gap on the unidentified prevalence of patients with obstruction in the US.

No follow-up studies have evaluated prevalence of clinically recognized oHCM in the US in recent years, and trends of oHCM prevalence over time using medical and pharmacy claims data is unknown. Understanding the true prevalence of clinically diagnosed patients with oHCM in the US population may improve the screening, identification and treatment of patients; specifically, those who do not seek specialty care for their disease at an HCM Center of Excellence. Therefore, we estimated the annual prevalence of clinically diagnosed oHCM in the US from 2016 to 2018, including by sex and age.

## Materials and Methods

### Data Source

This retrospective observational study used the MarketScan® Commercial Claims and Encounters Database from IBM Watson Health® (MarketScan), which contains de-identified, patient-specific data on reimbursed healthcare claims for employees, retirees, and their dependents of over 250 medium and large employers and health plans. Individuals included in the database are covered under private insurance plans; no Medicaid or Medicare data are included. These data cover approximately 28 million covered employees and family members per year. MarketScan is divided into subsections including: Inpatient Claims file, Outpatient Claims file, Outpatient Prescription Drug Claims file, Enrollment Information file, and RED BOOK™ Supplement.

### Analysis

**Figure 1:**
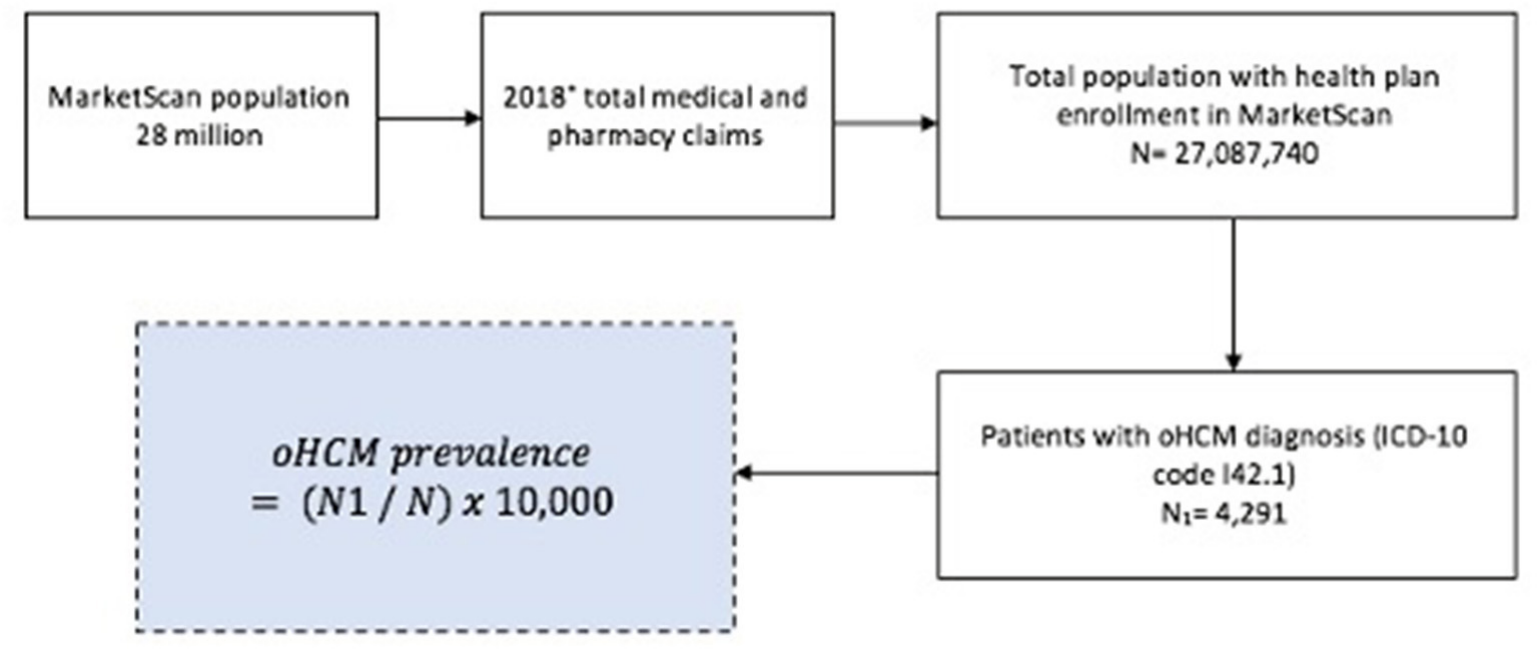
Flow diagram for US prevalence of oHCM in 2018. US prevalence of oHCM in 2018 from MarketScan. oHCM, obstructive hypertrophic cardiomyopathy; ICD- 10, International Classification of Diseases, Clinical Modification Ninth and Tenth. *Prevalence was calculated for all years from 2016–2018.

Analyses were undertaken with SAS, version 9.3 (SAS Institute Inc., Cary, NC, USA). Patients with clinically diagnosed oHCM were identified by the presence of at least 1 claim with a diagnosis of oHCM (ICD-10 code I42.1). The number of unique patients with clinically diagnosed oHCM was determined for each year, and the annual prevalence rate for diagnosed oHCM was defined as the number of patients diagnosed with the disease divided by the total number of health plan members at risk during the calendar year (Figure 1). Prevalence of oHCM was reported per 10,000 persons and stratified by sex and age group (<18, 18–34, 35–44, 45–54, 55–64, ≥65). Two population proportion tests addressed statistical significance of categorical variables. Tests were 2-sided and *P* < 0.05 was considered statistically significant. This study was approved by the Penn State College of Medicine Institutional Review Board.

## Results

**Figure 2:**
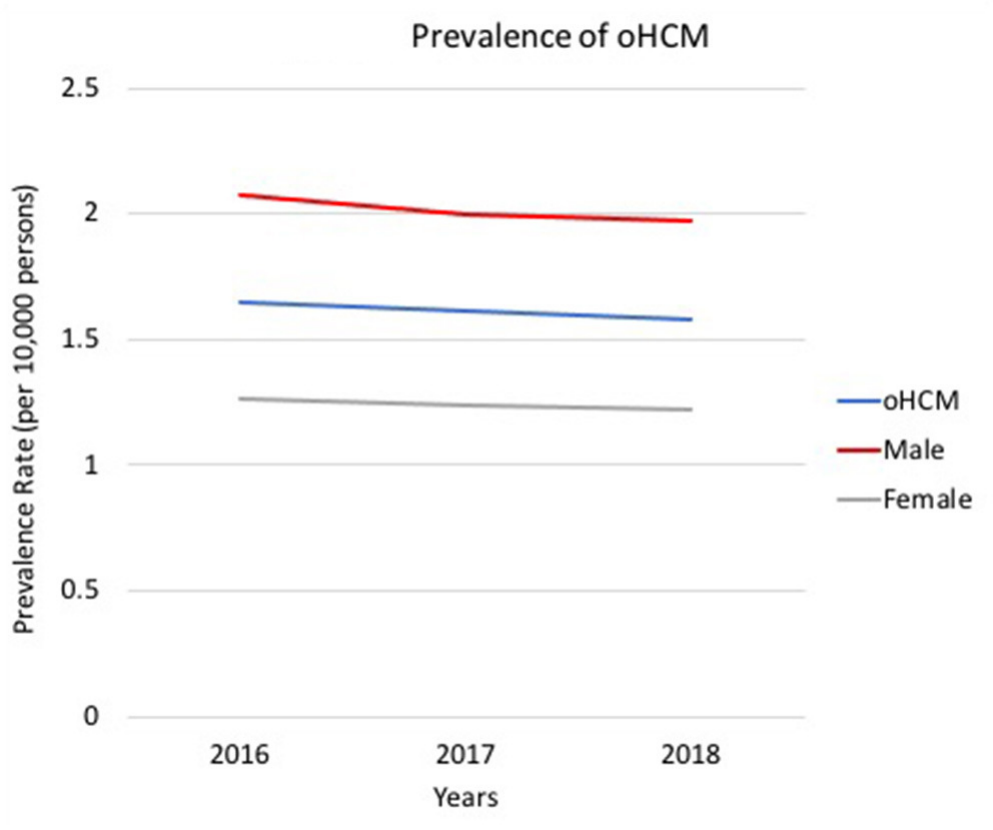
Prevalence of oHCM. Trends of oHCM per 10,000 persons in the US from 2016 – 2018, including by sex. oHCM, obstructive hypertrophic cardiomyopathy; US, United States.

In 2016, 4,612 unique patients had a claim with a clinical diagnosis of oHCM, resulting in an estimated oHCM prevalence of 1.65 per 10,000. Prevalence of oHCM in MarketScan shows the prevalence of oHCM remained similar in 2017 and 2018: 1.61 and 1.58, respectively (Table 1). Prevalence Rates of oHCM reports the prevalence of oHCM in 2016 was higher in males than in females (2.07 and 1.26, respectively) (Figure 2). Trends of prevalence by sex remained similar over the time-period and prevalence of oHCM in males remained higher than in females from 2017 to 2018 (*p* < 0.00001). While the overall prevalence of oHCM decreased slightly over the period, it increased with age.

**Table 1:** Prevalence of oHCM in MarketScan (per 10,000 persons).

	**Sex**				**Age Group (years)**						
**Year**	**oHCM**	**Male**	**Female**	***P*-value**	** <18**	**18–34**	**35–44**	**45–54**	**55–64**	**≥65**	***P*-value**
2016	1.65	2.07	1.26	<0.00001	0.36	0.62	1.21	2.43	4.82	2.36	<0.00001
2017	1.61	2.00	1.24	<0.00001	0.36	0.57	1.15	2.38	4.75	1.81	<0.00001
2018	1.58	1.97	1.22	<0.00001	0.31	0.54	1.12	2.37	4.84	2.73	<0.00001

*oHCM, obstructive hypertrophic cardiomyopathy*.

**Figure 3:**
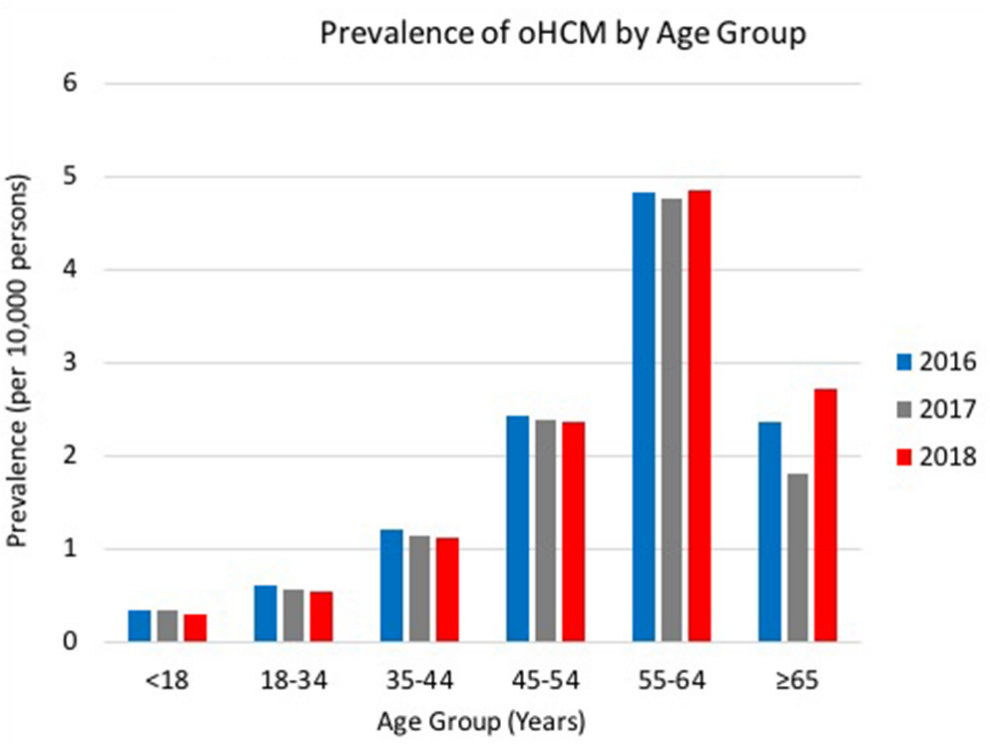
Prevalence of oHCM by Age Group. Trends of oHCM per 10,000 persons in the US by age group from 2016 – 2018. oHCM, obstructive hypertrophic cardiomyopathy; US, United States.

Prevalence of oHCM by Age Group shows an increased trend of oHCM with age over the time period, beginning with a 2016 oHCM prevalence by age: 0.36 (<18 years) to 2.36 (≥65 years) (*p* < 0.00001) (Figure 3). Prevalence of oHCM by age group in 2016 included 0.36 (<18 years), 0.62 (18–34 years), 1.21 (35–44 years), 2.43 (45–54 years), 4.82 (55–64 years), 2.36 (≥65 years). The figure shows consistent trends in oHCM by age group across the latter 2 years (*p* < 0.00001), with oHCM prevalence in 2018 being 0.31 (<18 years), 0.54 (18–34 years), 1.12 (35–44 years), 2.37 (45–54 years), 4.84 (55–64 years), 2.73 (≥65 years). Over the 3-year time period, the prevalence of oHCM remained the greatest in patients 55–64 years of age.

## Discussion

The objective of this study was to estimate the annual prevalence of clinically diagnosed oHCM in the US from 2016 to 2018, including by sex and age. To our knowledge, there is limited evidence on the use of real-world data to analyze prevalence of oHCM in the general US population, or trends of oHCM prevalence over time. Using a large, national database of private insurance claims, we found that the prevalence of oHCM was 1.65 per 10,000 individuals in 2016 and remained fairly stable over the 3-year time period. This is considerably lower than the rate of patients with obstruction found in a previous cardiac magnetic resonance-based (1 in 517 persons) (12) representing oHCM patients in the UK. These results provide unique data on trends of oHCM over time and stable rates of oHCM over a 3-year period may suggest the gap on identifying patients with oHCM in the US is not getting smaller.

There are many advantages of using claims data for research. Claims data allow for a large, diverse sample of patients treated in various care settings across all geographic regions in the US. The majority of previous studies analyzing patients with oHCM represent single- or multi-center Centers of Excellence, embodying patients in specialty cardiovascular settings receiving high quality care in specific geographic regions. Studies using claims data allow for longitudinal follow-up of patients to evaluate prevalence, demographics, clinical characteristics, outcomes, and patterns of care and changes over time. Additionally, because claims data are used for billing purposes, they are carefully reviewed for accuracy. Claims data are not limited to services provided at one or a few healthcare centers; therefore, capturing data from over 250 employers and health plans is more generalizable to privately insured patients in the US population.

A recent study using the UKBB encompassing 39,551 participants with cardiac magnetic resonance imaging aged 40–69 years across the UK between 2006 and 2010 analyzed cardiovascular phenotypes according to the presence of rare variants in sarcomere-encoding genes (12). de Marvao and colleagues found the prevalence of phenotypic HCM (defined as wall thickness ≥15 mm in the absence of hypertension and valve disease) was 0.19% (1 in 517) (12). This finding on prevalence from the UKBB is much greater than reported in our results (1.65 per 10,000 individuals). Similar to our study, the UKKB is a population-based cohort of patients with oHCM across the UK, whereas our data source (MarketScan) is generalizable to all patients with commercial health insurance in the US. However, a major difference is de Marvao and colleagues identify prevalence of oHCM using guideline recommended diagnostics using cardiac magnetic resonance (12). Patients in our cohort were defined as clinically diagnosed oHCM based on the presence of at least 1 medical claim with a diagnosis of oHCM (ICD-10 code I42.1). Our methods may not be reliable in the absence of patient level anatomic or genetic confirmation.

The only other study to report on prevalence of HCM (not specifying obstruction) in the US using medical and pharmacy claims was by Maron et al. (5). Similar to our methods, Maron et al. used a large proprietary claims database (Symphony Health Solutions) to identify clinically diagnosed HCM in 2013. Their analysis used ICD-9 diagnosis codes to report prevalence of all HCM types (HCM, oHCM, other HCM including non-obstructive) (5), whereas our analysis used ICD-10 diagnosis codes to capture only HCM patients with obstruction. They estimated an overall prevalence of 0.03% and found that prevalence of HCM was highest in the fifth decade of life, similar to our results. In addition, their cohort was comprised of 43% women (5), similar to our cohort of 40% women. Maron et al. (5) had some important advantages relative to our study. The Symphony Health Solutions database is larger, covering 160 million individual patients (vs. 28 million patients in MarketScan) in the US, and is comprised of all insurance types including Medicare and Medicaid (5). While individuals included in MarketScan are covered under private commercial insurance plans only (no Medicaid or Medicare), our study has unique advantages relative to previous investigations. Our analysis includes contemporary data over a 3-year time period, allowing us to analyze longitudinal trends in oHCM prevalence over time. Additionally, clinical disease identification utilized an updated reporting system of ICD-10 diagnosis codes, which are more specific and generally can be considered a more precise reporting of clinically diagnosed oHCM than previous ICD versions.

The opportunity to access a large national claims database provides a robust and updated presentation of the prevalence of patients with clinically diagnosed oHCM in the general US population. These results suggest that the majority of privately insured patients with oHCM are undiagnosed and are consistent with previous investigations that an overwhelming amount of HCM patients in the US are clinically unrecognized and the number of diagnosed HCM patients is significantly lower than the estimated prevalence of HCM in the general population. This study may reflect actual differences in the underlying prevalence and presentation of oHCM but may also suggest that oHCM is better recognized in families with a history of HCM and patients that have insurance to access specialty healthcare services, including contemporary cardiovascular imaging and genetic testing. The gap of clinical identification remains with patients in the general US population with unknown genetics and/ or cardiovascular medical history. This study reinforces the need for nationwide policies and screening strategies to improve the clinical identification of oHCM patients in the US.

## Limitations

There are several limitations of this analysis that are common in claims data. First, administrative data are primarily collected for billing and reimbursement purposes and may be subject to coding biases, inconsistencies, and missing data. Second, MarketScan includes only individuals with private insurance and does not include Medicaid or Medicare. Thus, the results may not be generalizable to patients with other types of health insurance, who are uninsured, or live outside the US. Third, the use of administrative claims data in this analysis relies on ICD-10 diagnosis codes, which does not include patient level anatomic or genetic confirmation. Fourth, this study did not include patients with non-obstructive HCM. We focused on oHCM in order to estimate the number of patients that are symptomatic and require contemporary treatments by experienced referral centers. Fifth, ICD-10 was not introduced until October 2015, limiting our ability to capture more than 3 full years of data. Sixth, we did not require an additional claim for appropriate drug or septal reduction therapy criteria in our methods which may further distinguish case identification of patients with obstructive HCM; however, this would exclude patients with obstruction that are asymptomatic and not receiving treatment and limit our ability to capture the true prevalence of all patients with obstructive HCM in the MarketScan. A major strength of the study, however, is the ability to observe the healthcare service use of a large, national sample and accurately provide a robust presentation of prevalence trends of patients diagnosed with oHCM in the US.

## Conclusion

In a large medical and pharmacy claims analysis, the prevalence of clinically recognized oHCM was lower than prevalence rates from echocardiography-based studies, and it remained stable from 2016 to 2018. The prevalence of oHCM was consistently slightly higher in male patients over the study period, with the highest prevalence of oHCM in patients age 55–64 years. These new data support previous studies suggesting that the majority of patients with oHCM are undiagnosed in the US, supporting the need for new strategies to close the gap on unrecognized oHCM. Identifying the true prevalence of clinically diagnosed patients with oHCM may improve early identification and treatment of patients and these results reinforce the imperative need for new policies and future research to improve the clinical identification of oHCM patients in the US.

## Data Availability Statement

The original contributions presented in the study are included in the article/Supplementary material, further inquiries can be directed to the corresponding author.

## Ethics Statement

This study was approved by the Penn State College of Medicine Institutional Review Board.

## Author Contributions

MB and DL conducted the data analysis. MB wrote the first draft of the article with input from the other authors. All authors were involved in the study design, contributed to the interpretation of the results, writing or revision of the manuscript, and approved the decision to submit the article for publication.

## Funding

The proposed study was funded and being conducted by Penn State University Department of Public Health Sciences.

## Conflict of Interest

The authors declare that the research was conducted in the absence of any commercial or financial relationships that could be construed as a potential conflict of interest.

## Publisher's Note

All claims expressed in this article are solely those of the authors and do not necessarily represent those of their affiliated organizations, or those of the publisher, the editors and the reviewers. Any product that may be evaluated in this article, or claim that may be made by its manufacturer, is not guaranteed or endorsed by the publisher.

## Abbreviations

HCM: hypertrophic cardiomyopathy
US: United States
oHCM: obstructive hypertrophic cardiomyopathy
ICD-10, International Statistical Classification of Disease and Related Health Problems diagnosis code: version 10.
